# Selection Signatures and Genetic Divergence in Hotan Black and F2 Yeonsan Ogye Chickens

**DOI:** 10.3390/ani16101511

**Published:** 2026-05-15

**Authors:** Nursat Turxunjan, Yuxuan Liu, Min Liu, Tianci Liu, Wenqiang Hou, Huie Wang

**Affiliations:** 1 College of Animal Science and Technology, Tarim University, Alar 843300, China; 15559464033@163.com (N.T.); 15620421068@163.com (Y.L.); m18705096460@163.com (M.L.); 13633380110@163.com (T.L.); 15518572135@163.com (W.H.); 2 Key Laboratory of Livestock and Grass Resources Utilization Around Tarim, Ministry of Agriculture and Rural Areas (Co-Construction by Ministries and Provinces) & Construction Corps, Alar 843300, China

**Keywords:** population structure, selection signals, Hotan Black Chicken, Yeonsan Ogye, F2 hybrid, population genomics

## Abstract

Local chicken breeds have long been subjected to unique geographical isolation and extreme environmental pressures, which have led to significant environmental adaptation. This provides a valuable opportunity to explore the mechanisms of adaptive evolution in domestic chickens and to develop rare genetic resources. However, the molecular genetic basis of these local breeds remains inadequately studied. In this research, we systematically compared the population genetic structure and selection signals between the Hotan Black Chicken (HT) from Xinjiang and the F2 hybrid population of Yeonsan Ogye × White Leghorn chickens (OLF) based on medium-density SNP chip data. Principal Component Analysis (PCA), Neighbor-Joining (NJ) phylogenetic trees, and ancestry inference methods revealed significant genetic differentiation between the two populations. HT exhibited a relatively homogeneous genetic background, while the F2 population showed a more complex genomic structure. By combining a sliding window approach with population differentiation index (*F_ST_*), nucleotide diversity (π), and the ratio log2(π_HT/π_OLF), we identified selection signal regions across the whole genome, with 99 candidate genes for the OLF population and 36 for the HT population. The candidate regions in the HT population were enriched in pathways related to environmental adaptation, including cell adhesion, neurodevelopment, and immune regulation, while the F2 population showed significant enrichment in PI3K/AKT signaling and protein phosphorylation pathways related to production performance. This study not only elucidates the adaptive genomic features of local chicken breeds in extreme environments but also provides a genomic basis for the conservation and molecular breeding of local chicken genetic resources.

## Simple Summary

This study compares the genetic structure of Hotan Black Chicken (HT) and an F2 hybrid population of Yeonsan Ogye × White Leghorn chickens (OLF) using SNP chip data. HT shows stronger environmental adaptations, while OLF exhibits selection signals related to production performance. These findings provide valuable insights for the conservation and breeding of local chicken breeds.

## 1. Introduction

The chicken (*Gallus gallus domesticus*) is one of the most important poultry species worldwide, extensively used in meat and egg production, and plays a central role in global agriculture and animal husbandry [1,2,3]. In addition to highly commercialized chicken breeds, many local chicken breeds are still preserved around the world. These breeds have developed unique phenotypic traits and genetic backgrounds under long-term geographical isolation, climate pressures, and variations in breeding management practices. Compared to commercial breeds, local chicken breeds typically retain a greater genetic diversity, which provides significant potential breeding value in terms of environmental adaptability, stress resistance, and specific production traits [4]. Therefore, a thorough analysis of the genetic differentiation patterns in local chickens, along with the identification of their selection signals, is crucial for understanding the adaptive evolutionary processes of domestic chickens and for the conservation and rational use of local chicken genetic resources.

The Hotan Black Chicken (HT) is one of the local chicken breeds distributed in the Xinjiang region of China, characterized by an arid environment, large diurnal temperature fluctuations, and relatively extensive farming conditions [5,6]. Previous studies have shown that local chickens in Xinjiang generally exhibit strong environmental adaptability. The Hotan Black Chicken, in particular, demonstrates significant advantages in disease resistance, tolerance to rough feeding, and heat resistance, suggesting that its genome may harbor genetic variations associated with environmental adaptation and stress resistance. However, despite initial attention to the ecological adaptation features of the Hotan Black Chicken, systematic studies on its population genetic structure, genetic differentiation, and potential selection signals remain limited, and its molecular genetic foundation at the genomic level requires further elucidation. In population comparison studies, experimental hybrid populations are widely used for population genetic analysis and selection signal research due to their well-defined genetic backgrounds and clear sources of variation [7,8]. The F2 hybrid population of Yeonsan Ogye × White Leghorn chickens combines the genetic components of local and commercial chicken breeds, providing a valuable comparative framework for studying population differentiation patterns under different genetic backgrounds [9]. By comparing the genomes of local chicken breeds and hybrid populations, significant genomic regions showing differentiation or diversity changes can be identified, offering valuable insights into the genetic features and breeding potential of local chickens.

Methodologically, medium-density SNP chips have been widely used in poultry population structure analysis, genetic diversity assessment, and whole-genome selection signal scanning due to their controlled cost, high repeatability, and suitability for analysis of moderately sized samples [10,11]. Based on SNP chip data, the population differentiation index (*F_ST_*) quantifies the degree of genetic differentiation between populations, while nucleotide diversity (π) reflects the level of genetic variation within populations [12]. Chen et al. used *F_ST_* analysis combined with θπ in a comparative study of Luning chicken and seven other chicken breeds, identifying key genes involved in melanin deposition, such as *ATP5E*, *EDN3*, and *ELMO2*. The sliding window strategy was employed for whole-genome scanning, effectively reducing noise from individual loci and identifying potential selection signals within continuous genomic regions [13].

Based on the aforementioned background, this study focuses on the Hotan Black Chicken (HT) and the F2 hybrid population of Yeonsan Ogye × White Leghorn chickens (OLF × WL F2), utilizing SNP chip genotype data for population genomics analysis. First, population structure and genetic relationships were assessed using Principal Component Analysis (PCA), Neighbor-Joining (NJ) phylogenetic trees, and ancestry inference methods. Next, a sliding window strategy was employed to jointly scan *F_ST_*, nucleotide diversity (π), and the ratio log2(π_HT/π_OLF) across the whole genome, identifying candidate regions with significant genetic differentiation or abnormal diversity changes. Finally, gene annotation and functional enrichment analyses were performed on these candidate regions to reveal potential selection signals in the Hotan Black Chicken genome, providing a genomic basis for the conservation and molecular breeding of local chicken genetic resources.

## 2. Materials and Methods

### 2.1. Ethics Statement

This study involved the collection and use of animal samples and strictly adhered to the animal ethics and experimental guidelines of Tarim University. The animal study protocol was approved by the Institutional Animal Care and Use Committee of Tarim University (protocol code 2024064, approved on 4 June 2024).

### 2.2. Study Subjects and Sample Collection

This study selected two chicken breeds: Hotan Black chicken (HT) and Yeonsan Ogye chicken (OLF). The HT samples were collected from 144 individuals at the Tarim University Experimental Station, while the OLF samples were obtained from a publicly available dataset on Figshare (https://doi.org/10.6084/m9.figshare.21620856.v1), which includes data from 144 individuals. The collected samples were peripheral blood, which was used for genomic DNA extraction.

### 2.3. DNA Extraction and Chip Genotyping

Genomic DNA was extracted from chicken peripheral blood samples using the TIANamp Genomic DNA Kit (TIANGEN, Beijing, China). DNA concentration and purity were assessed using NanoDrop spectrophotometer (Thermo Fisher Scientific, Wilmington, DE, USA) and Qubit fluorometer (Invitrogen, Carlsbad, CA, USA), with samples having an OD 260/280 ≥1.8 being further validated for quality via 1% agarose gel electrophoresis. All DNA samples were stored at −20 °C until further analysis. Genotyping was performed using the Illumina Chicken 60K SNP BeadChip (Illumina, San Diego, CA, USA). Clustering of raw signals and genotype calling were carried out following the standard protocol of Illumina’s GenomeStudio (V2.0.5) software, and the data were subsequently converted to PLINK format for downstream genetic analysis.

### 2.4. Genotype Data Quality Control

The merged genotype data were subjected to quality control (QC) using PLINK (v1.9). First, SNPs with a missing rate greater than 10% were excluded using the geno 0.1 filter to ensure data completeness. Next, SNPs with a minor allele frequency (MAF) lower than 5% were removed using the maf 0.05 filter to enhance the reliability of the data’s variation information. To eliminate biases caused by population structure or genotype errors, SNPs significantly deviating from the Hardy–Weinberg equilibrium were excluded using the hwe 1 × 10^−6^ filter. These filtering steps resulted in the selection of high-quality SNPs for subsequent population genetic analysis.

### 2.5. Population Structure

To evaluate the genetic structure and potential population admixture between Hotan Black Chicken (HT) and the F2 hybrid population of Yeonsan Ogye × White Leghorn chickens (OLF), this study employed Principal Component Analysis (PCA), Neighbor-Joining (NJ) phylogenetic tree construction, and sparse Non-negative Matrix Factorization (sNMF) methods to estimate the ancestry composition of individuals from different ancestral populations.

First, PCA was performed on the quality-controlled data using PLINK software. PCA was based on a variance-standardized relationship matrix, aiming to display the separation between populations and the potential population structure by calculating the major genetic differences among samples. Next, the NJ matrix was computed using the VCF2D is tool (https://github.com/BGI-shenzhen/VCF2Dis, accessed 10 December 2025), and a phylogenetic tree was constructed based on this matrix. The NJ tree was generated using the FastME software (v2.1.6.1) (http://www.atgc-montpellier.fr/fastme/, accessed 10 December 2025), which effectively reveals the genetic relationships and evolutionary history of the samples through a distance matrix-based tree-building algorithm. The phylogenetic tree was then visualized and annotated using the iTOL platform (https://itol.embl.de/, accessed 10 December 2025) to further clarify the genetic distances and relationships between different populations. Finally, the sNMF method was applied to infer the ancestry composition of the HT and OLF populations [14]. The sNMF method estimates the proportion of each individual in pre-defined K ancestral clusters and uses the cross-entropy criterion to select the best K value (ranging from 2 to 10). This analysis effectively distinguishes the genetic admixture patterns between populations and reveals the structural differences among them.

### 2.6. Selection Signals

The analysis of selection signals aims to identify potential selection pressures experienced by Hotan Black Chicken (HT) and the F2 hybrid population of Yeonsan Ogye × White Leghorn chickens (OLF). To achieve this, the study combined population differentiation index (*F_ST_*), nucleotide diversity (π), and their ratio log2(π_HT/π_OLF) for a whole-genome scan to identify significant selection signal regions.

The population differentiation index (*F_ST_*) is a commonly used metric to measure genetic differences between populations. The *F_ST_* calculation is based on the formula proposed by Weir and Cockerham, which quantifies the degree of genetic differentiation between two populations. The specific calculation formula is:

(1) $$F_{ST}=\frac{{\left(p_{A}-p_{B}\right)}^{2}}{p_{A}(1-p_{A})+p_{B}(1-p_{B})}$$

In this formula, $p_{A}$ and $p_{B}$ represent the allele frequencies at a particular SNP site for the HT and OLF populations, respectively. The *F_ST_* value ranges from 0 to 1, with higher values indicating greater genetic differentiation between populations. In this study, the *F_ST_* values were calculated using VCF tools, with a sliding window approach applied to scan the genome-wide. The window size was set to 50 kb, with a step size of 25 kb. Based on the filtering criteria, the top 5% of *F_ST_* values across the genome were selected as significant genetic differentiation regions, which are likely to have undergone selection pressures.

To further assess the level of genetic variation within populations, nucleotide diversity (π) was calculated. Nucleotide diversity represents the degree of genetic variation among different genotypes within a population. The calculation formula is as follows:

(2) $$\pi =\frac{1}{n(n-1)}\sum_{i<j}d_{ij}$$

In this formula, n represents the sample size, and $d_{ij}$ is the genotype difference between sample i and sample j at a particular SNP site. Higher nucleotide diversity (π) indicates greater genetic variation within a population. This study also calculated the ratio log2(π_HT/π_OLF) to assess the relative diversity difference between the HT and OLF populations in the same region. A significant increase or decrease in this ratio suggests that the region may have undergone selection pressure. To identify potential selection regions, this study selected the top and bottom 2.5% percentiles of the log2(π_HT/π_OLF) ratio as candidate regions.

### 2.7. Candidate Region Annotation and Functional Analysis

To improve the accuracy of selection signal analysis, this study performed an intersection analysis of candidate windows selected based on *F_ST_* and log2(π_HT/π_OLF), ultimately identifying genomic regions that exhibit both significant population differentiation and abnormal diversity changes. This approach effectively identifies genomic regions likely under selection. Genome annotation was based on the chicken reference genome (e.g., GRCg6a version), and gene annotation for the candidate regions was performed using BTools for interval overlap analysis [15].

### 2.8. Functional Enrichment of Candidate Genes

GO functional enrichment analysis was performed using the DAVID website (https://davidbioinformatics.nih.gov/, accessed 10 December 2025). First, gene set functional annotation was conducted using the online tool provided by DAVID, with the species set to chicken (*Gallus gallus*). Gene Ontology (GO) analysis was then performed on these genes via the DAVID platform to identify significantly enriched terms related to biological processes, molecular functions, and cellular components. This analysis helps to determine the genes and pathways that may play a key role in the selection signal regions.

## 3. Results

After merging the two analysis populations and performing quality control, a total of 39,443 high-quality SNPs were retained (Supplementary Materials Table S1).

### 3.1. Population Structure Analysis

**Figure 1:**
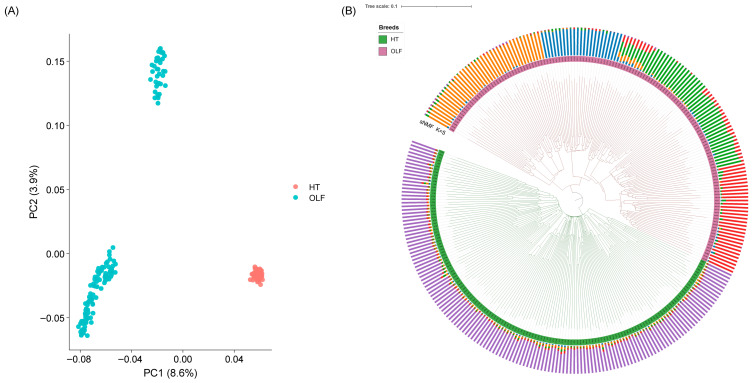
Population Structure Analysis Results. (**A**) Principal Component Analysis (PCA) plot showing the distribution of HT and OLF populations along the PC1 (8.6%) and PC2 (3.9%) axes. Different colors represent the two populations: HT is marked in orange, and OLF is marked in light blue. (**B**) Phylogenetic tree constructed using the Neighbor-Joining (NJ) method, combined with the ancestry composition plot from the sparse Non-negative Matrix Factorization (sNMF, K = 5) analysis. The inner circle represents the NJ tree, illustrating the genetic distance relationships among the samples; the outer circle displays the sNMF (K = 5) results, indicating the ancestry assignment of each sample in different genetic clusters. The population assignment of each sample is represented by the color bars in the outer circle.

Firstly, the results of principal component analysis (PCA) showed that the HT and OLF populations were separated along the PC1 axis, indicating subtle genetic differentiation between the two breeds. In addition, the OLF population was divided into two distinct clusters along the PC2 axis. Although PC1 explained 8.6% of the total variance and PC2 explained 3.9%, differentiation within the OLF population was supported to some extent (Figure 1A).

Second, a lineage inference analysis using sparse non-negative matrix factorization (sNMF) found that the best population structure was obtained when K = 5 (Supplementary Materials Figure S1). At this value of K, OLF populations exhibit a complex mix of lineages, reflecting a high degree of genetic heterogeneity within populations. In contrast, the HT population exhibited a relatively simple ancestral composition, further supporting its more purified genetic background, whereas the OLF population exhibited a more complex genome structure.

Finally, the neighborhood join (NJ) tree based on the genetic distance matrix was constructed, and the results showed that there was a clear separation between HT and OLF populations, which further confirmed their significant genetic differentiation. The OLF population showed a more complex branching structure, which is consistent with the results of PCA and sNMF, suggesting that the OLF population may have undergone genetic recombination and mixing over multiple generations (Figure 1B).

### 3.2. Selection Signal Analysis

**Figure 2:**
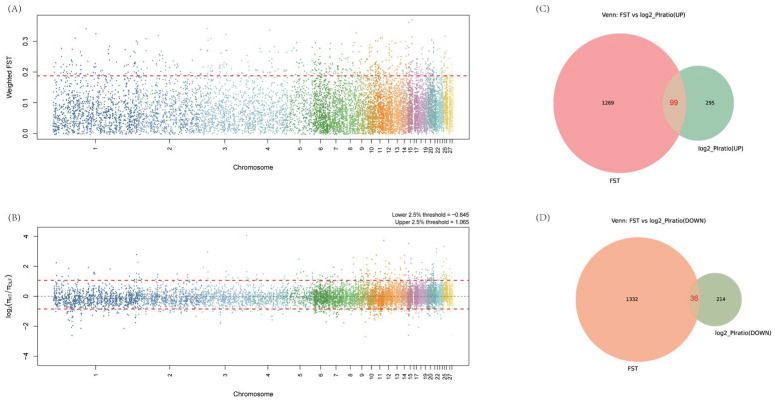
Manhattan Plots and Venn Diagrams of *F_ST_* and log2(π_HT/π_OLF) Ratio. (**A**) *F_ST_* Manhattan plot showing genetic differentiation between the HT and OLF populations. The red dashed line represents the top 5% *F_ST_* value threshold, indicating regions potentially affected by selection pressure. (**B**) Manhattan plot of the log2(π_HT/π_OLF) ratio showing the relative diversity differences between the HT and OLF populations across different genomic regions. The red dashed line represents the upper and lower 2.5% percentiles of the ratio, highlighting regions potentially influenced by selection pressure. (**C**) Venn diagram showing the intersection of SNPs in the top 5% of *F_ST_* values and the top 2.5% regions of the log2(π_HT/π_OLF) ratio, resulting in 99 candidate genes. (**D**) Venn diagram showing the intersection of SNPs in the top 5% of *F_ST_* values and the bottom 2.5% regions of the log2(π_HT/π_OLF) ratio, resulting in 36 candidate genes.

To identify selection signals between the HT and OLF populations, this study combined *F_ST_* values and the log2(π_HT/π_OLF) ratio, using a sliding window approach to scan the genome-wide. The *F_ST_* Manhattan plot shows that multiple genomic regions exhibit significant genetic differentiation between HT and OLF populations (Figure 2A). Based on the top 5% *F_ST_* value threshold, we identified 1368 genes under selection pressure (Supplementary Materials Table S2). The log2(π_HT/π_OLF) Manhattan plot reveals the diversity differences between the HT and OLF populations across different genomic regions (Figure 2B). Using the upper and lower 2.5% percentiles of the log2(π_HT/π_OLF) ratio, we selected 394 and 250 genes from the two populations, respectively (Supplementary Materials Table S3). When the ratio was significantly increased, 99 intersecting genes indicated that selection pressure may have occurred in the OLF population (Figure 2C), whereas 36 intersecting genes suggest selection in the HT population (Figure 2D).

### 3.3. GO Functional Enrichment Analysis

In the F2 hybrid population (OLF), due to its higher genetic diversity, certain advantageous traits related to production performance, meat quality, flavor, and disease resistance may be present (Supplementary Materials Table S4). GO functional enrichment analysis revealed several significant pathways that may reflect the advantageous traits of the Yeonsan Ogye × White Leghorn F2 population. Specifically, pathways related to metabolism, cell repair, and immunity highlighted the key roles of the F2 population in various biological processes.

**Figure 3:**
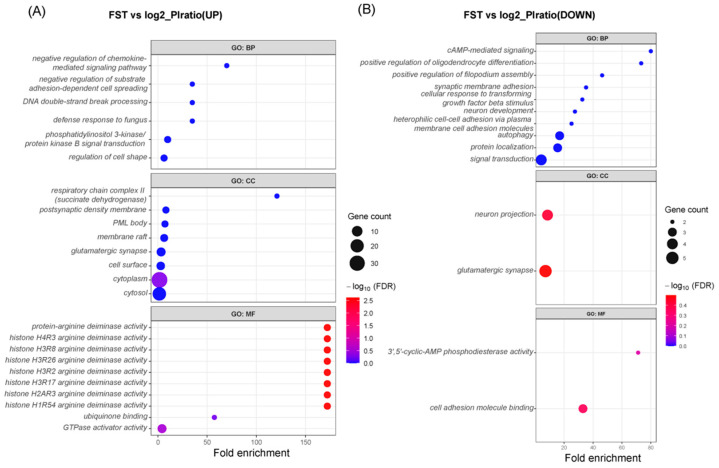
GO Functional Enrichment Analysis of *F_ST_* and log2(π_HT/π_OLF) Ratio. (**A**) Enriched pathways in the top 2.5% regions of *F_ST_* and log2(π_HT/π_OLF) ratio. GO analysis revealed significantly enriched biological processes (BP), cellular components (CC), and molecular functions (MF), including Cell adhesion molecule binding (GO:0050839), Neuronal projection (GO:0043005), and Glutamatergic synapse (GO:0098978). These pathways suggest that genes in these regions may play important roles in intercellular interactions, neurodevelopment, and immune response. These regions are primarily associated with selection pressure in the OLF population, showing significant genetic differentiation. (**B**) Enriched pathways in the bottom 2.5% regions of *F_ST_* and log2(π_HT/π_OLF) ratio. GO analysis revealed significantly enriched biological processes (BP), cellular components (CC), and molecular functions (MF), including cAMP-mediated signaling (GO:0023051), Neuronal projection (GO:0043005), and Glutamatergic synapse (GO:0098978). These genes are related to neurodevelopment and cell signal transduction. The size of the points in the figure represents the number of enriched genes, and the color intensity reflects statistical significance (FDR values).

First, the enrichment of the Phosphoinositide 3-kinase/Protein kinase B (PI3K/AKT) signaling pathway (GO:0043491) indicates that the F2 population plays an important role in cell signaling, potentially involved in growth and metabolism, which contributes to the improved production performance of the population. Second, the enrichment of the Protein phosphorylation (GO:0016310) and Protein binding (GO:0005515) pathways suggests that these genes are closely related to cell function, protein interactions, and growth regulation, further supporting the advantages of the Yeonsan Ogye × White Leghorn F2 hybrid population in terms of growth rate and production efficiency (Figure 3A).

In contrast, candidate genes selected from the bottom 2.5% of the log2(π_HT/π_OLF) ratio regions revealed 15 significantly enriched pathways related to environmental adaptability, immune response, and cell repair (Supplementary Materials Table S5). First, the enrichment of the Cell adhesion molecule binding (GO:0050839) and Neuronal projection (GO:0043005) pathways suggests that these genes may be involved in intercellular interactions, neurodevelopment, and immune system regulation, providing a potential biological foundation for the survival of the HT population in extreme environments. Additionally, the enrichment of Glutamatergic synapse (GO:0098978) and Neuronal projection (GO:0043005) further supports the adaptive role of HT in neurodevelopment, aiding its neurological function in harsh environments.

In terms of biological processes, the enrichment of Autophagy (GO:0006914) suggests that the HT population may enhance its survival ability in arid and extreme temperature fluctuation environments by removing damaged cell components or responding to cellular stress. The enrichment of Protein localization (GO:0008104) indicates that these genes play a role in intracellular material transport and repair mechanisms, optimizing the HT population’s adaptation to environmental stress. At the molecular function level, the enrichment of Protein phosphorylation (GO:0016310) and Protein binding (GO:0005515) reveals the potential roles of HT in signal transduction and molecular interactions, further supporting its adaptability and immune tolerance (Figure 3B).

## 4. Discussion

### 4.1. Genetic Structure and Evolutionary Background of Hotan Black Chicken

The results of principal component analysis (PCA) indicated that genetic differentiation may exist between the Hotan Black Chicken (HT) and the Yanshan Wuxi × White Leghang chicken (OLF) F2 cross population (Figure 1A). Although PCA showed limited variance explained by PC1 and PC2, subtle population differentiation could still be observed. The F2 OLF population showed a complex substructure due to recombination, whereas the HT population was relatively homogeneous. Given the low explained variance, phylogenetic trees constructed by Neighbor-Joining (NJ) methods further support these findings.

Sparse non-negative matrix factorization (sNMF) analysis further revealed that the HT population had a relatively simple lineage composition, while the OLF population exhibited a more complex mixed lineage pattern (Figure 1B). This result is consistent with the PCA analysis, which verifies the complexity of the genetic structure of the OLF population.

SNPs with intermediate minor allele frequencies were prioritized to improve genomic resolution and reduce bias in selection detection. Nevertheless, some genomic regions may remain underrepresented due to chip density limitations [16].

### 4.2. Adaptive Evolution of Hotan Black Chicken Population

The selection signal analysis based on whole-genome scanning revealed that the Hotan Black Chicken (HT) population exhibits significant genetic differentiation and low diversity in multiple genomic regions, indicating that these regions may have undergone strong selective sweeps during long-term adaptation to the extreme environmental conditions of arid, high-temperature, and large diurnal temperature fluctuations in Xinjiang (Figure 3B). Similar selection signals have been observed in other local chicken populations under varying altitudinal environmental pressures, particularly in pathways related to energy metabolism, immune response, and signal regulation [17].

The candidate selection regions in the HT population were enriched in functions such as GO:0050839 and GO:0043005, which are involved in cell signaling, intercellular interactions, and neurodevelopment [18,19,20]. These pathways may reflect the genetic optimization of cell structural homeostasis and neural regulation mechanisms in response to extreme environmental pressures in the HT population.

Additionally, the enrichment of biological processes such as GO:0006914 suggests that the HT population plays a critical role in maintaining neuronal homeostasis and responding to stress, which may be crucial for survival in extreme environments [21,22]. At the molecular function level, enriched terms such as Protein phosphorylation and Protein binding reveal the involvement of signal transduction and molecular interaction networks in the HT population’s selection signals. These mechanisms enable the HT population to rapidly adjust cellular responses and metabolic states under various environmental stimuli. These enriched terms are consistent with pathways related to environmental adaptation found in adaptive genomics studies, indicating that adaptive evolution often involves complex signal regulation and homeostasis maintenance mechanisms [17].

Overall, the enrichment results of candidate selection genes in the HT population reflect its adaptive evolutionary traits under complex environmental conditions such as drought and large temperature fluctuations.

### 4.3. Production Trait Advantages of the Yeonsan Ogye × White Leghorn F2 Hybrid Population

Unlike the environmental adaptation selection in the HT population, the Yeonsan Ogye × White Leghorn F2 hybrid population (OLF) exhibits selection signals related to production traits. The enrichment results from the top 2.5% regions of the log2(π_HT/π_OLF) ratio indicate that the OLF population is significantly enriched in the GO:0043491 (Figure 3A). This pathway plays a central role in cell proliferation, energy metabolism, and growth regulation, significantly impacting production traits such as meat quality, growth rate, and feed efficiency [23,24,25]. The activation of the PI3K/AKT signaling axis likely enhances tissue growth and energy utilization efficiency by regulating cell growth and metabolic pathways, providing the molecular foundation for the faster growth rate and improved production performance observed in the F2 population [26,27,28,29].

Additionally, the significant enrichment of (GO:0016310), a key regulatory mechanism in intracellular signal transduction cascades, further indicates the genetic optimization of the OLF population in signal regulation and metabolic responses (Figure 3A). Phosphorylation modifications directly affect various enzymes and receptor proteins, influencing physiological processes closely related to production traits, such as muscle development, protein synthesis, and cell cycle regulation [30].

## 5. Conclusions

This study reveals significant differences in genetic differentiation and adaptive evolution between Hotan Black Chicken (HT) and the Yeonsan Ogye × White Leghorn F2 hybrid population (OLF). The HT population exhibits selection signals related to cell signaling, neurodevelopment, and immune regulation, reflecting its environmental adaptability. In contrast, the OLF population shows advantages in production traits, particularly with enrichment in the PI3K/AKT signaling pathway and protein phosphorylation pathways. These findings provide important genomic insights for the conservation and molecular breeding of local chicken genetic resources.

## Data Availability

The original contributions presented in the study are included in the article/Supplementary Materials; further inquiries can be directed to the corresponding author.

## Supplementary Materials

The following supporting information can be downloaded at: https://www.mdpi.com/article/10.3390/ani16101511/s1, Supplementary Table S1: SNP-related information. Supplementary Table S2: Results of *F_ST_* analysis. Supplementary Table S3: Results of log2(π_HT/π_OLF) analysis. Supplementary Table S4: GO functional enrichment results for candidate genes in the OLF population. Supplementary Table S5: GO functional enrichment results for candidate genes in the HT population. Supplementary Figure S1. Elbow plot (K vs. Cross-Entropy).
