# Supplementary figures and images for: Selection Signatures and Genetic Divergence in Hotan Black and F2 Yeonsan Ogye Chickens
Source: Animals (Basel). 2026 May 15;16(10):1511. doi: 10.3390/ani16101511 (PMC13203631; doi:10.3390/ani16101511)

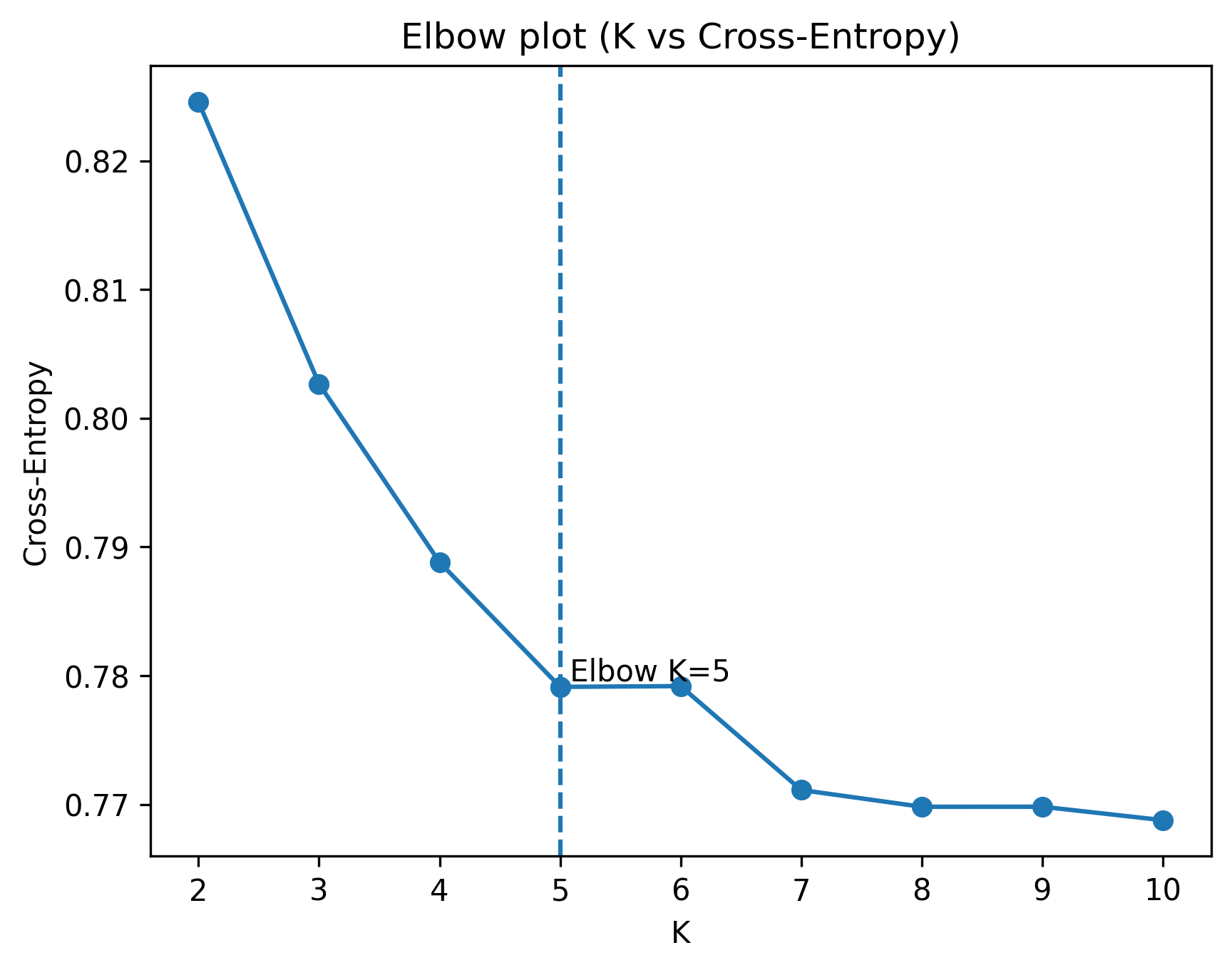

Supplement: Supplementary file 1 [file animals-16-01511-s001.zip › Supplementary Figure S1.png]
